# From need to action: exploring the motivations of women entrepreneurs through the triple-driver framework of women’s entrepreneurship

**DOI:** 10.3389/fpsyg.2026.1932247

**Published:** 2026-09-29

**Authors:** Ligat Shalev

**Affiliations:** School of Public Health, Hebrew University, Jerusalem, Israel

**Keywords:** ecosystem drivers, entrepreneur, organizational innovation, personal drivers, relational drivers, women

## Abstract

**Introduction:**

Women’s entrepreneurship has received increasing scholarly attention in recent years; however, research has largely overlooked women entrepreneurs whose ventures are specifically dedicated to addressing the needs of other women. Existing conceptualizations of women’s entrepreneurial motivations are primarily theoretical and have not been extensively validated using empirical data.This study aimed to explore the motivations of women entrepreneurs who established ventures addressing women’s needs and to develop an empirically grounded framework for understanding these motivations.

**Methods:**

A qualitative design was employed, involving in-depth semi-structured interviews with 60 Israeli women entrepreneurs. Data were analyzed using explanatory content analysis, guided by the competency-based framework.

**Results:**

The findings resulted in the development of the Triple-Driver Framework of Women’s Entrepreneurship, comprising three interrelated domains: personal drivers (e.g., lived experience and personal passion, justice-oriented values, autonomy), relational drivers (family and friends, role models), and ecosystem drivers (recognition of unmet societal needs, institutional support).

**Discussion:**

This study advances the literature by proposing an empirically grounded framework that broadens the theoretical understanding of women-centered entrepreneurship. The findings offer practical implications for policymakers and organizations seeking to foster women’s entrepreneurship and provide a foundation for future quantitative validation and cross-cultural research. For policymakers and organizations seeking to support women’s entrepreneurship, the Triple-Driver Framework provides a roadmap for understanding the personal, relational, and ecosystem-level pathways through which women are motivated to initiate and develop ventures. This understanding can inform the development of support efforts that are better aligned with women entrepreneurs’ motivations. For women entrepreneurs themselves, recognizing these motivational drivers may provide a clearer understanding of the factors shaping their entrepreneurial actions, helping them more intentionally define, direct, and develop their initiatives.

## 1 Introduction

### 1.1 Entrepreneurial motivations

Understanding entrepreneurial motivation is important because it shapes venture creation, entrepreneurial goals, and the value entrepreneurs seek to create (Abecassis-Moedas et al., 2021; Caliendo et al., 2023; Hossinger et al., 2023). Entrepreneurial motivation arises from multiple sources. One central dimension is personal motivation, which may reflect a desire for autonomy, greater control over work schedules and decision-making, or the pursuit of personal achievement (Caliendo et al., 2023; Van Gelderen et al., 2020). Another important source of entrepreneurial motivation is social and environmental motivation. This encompasses a desire to contribute to others, address community needs, and generate positive social impact (Bacq and Alt, 2018; Chaabane and Jarbou, 2025). It also includes responsiveness to external conditions, such as institutional frameworks, emerging market opportunities, and access to critical resources (Ebrahimi, 2026; Tong et al., 2026). Entrepreneurial motivation, whether personal, social, or environmental, can be conceptualized and categorized in different ways.

### 1.2 Self- and other-oriented entrepreneurial motivations

One axis on which entrepreneurial motivation can be categorized relates to whether it is primarily self- or other-oriented- in other words, the underlying motives. Self-oriented motivation reflects personal aspirations, such as the desire for autonomy, achievement, power, opportunity recognition, or a passion for innovation (Mascena Barbosa and Dumont, 2024; Ruskin et al., 2016). In contrast, other-oriented motivation refers to the desire to help others, address social or community needs, and create positive social impact (Ruskin et al., 2016; Yahyaoui et al., 2023). A third category, blended motivation, describes entrepreneurial activity driven by a combination of self-oriented and other-oriented motives (Fauchart and Gruber, 2011). Empirical research in social entrepreneurship suggests that many entrepreneurs experience a mix of personal and prosocial motives, rather than acting from a single dominant driver (Müller et al., 2026; Sadílek et al., 2022). Although there is a relationship between the type of entrepreneurial motivation and the type of organization established- for example, a for-profit organization versus a non-profit organization- the direction of this relationship is difficult to determine. This complexity highlights the importance of examining entrepreneurial motivations in relation to the specific populations entrepreneurs seek to serve and the forms of initiatives they develop.

### 1.3 Different types and forms of initiatives

Another axis on which entrepreneurial efforts can be categorized is the degree to which they have a commercial or a social focus. Commercially-focused initiatives are typically characterized by a primary focus on economic value creation (Li and Setiawan Sanusi, 2023). This includes profit generation, market growth, and competitive advantage, although these efforts may also incorporate personal and non-economic motives (Audretsch and Thurik, 2003; Sarman et al., 2025). In contrast, social initiatives are defined by their central commitment to addressing social or environmental challenges (Jareh, 2025; Polimetla, 2026). In these initiatives, creating value for others is the primary objective, while financial sustainability often serves as a means rather than an end (Pacheco et al., 2025; Phillips et al., 2015). However, as with the distinction between self- and other-oriented motivations, this distinction is not absolute, as many initiatives operate along a continuum, combining both economic and social motivations in hybrid or “blended” forms (Aveling et al., 2023; Ostertag et al., 2021). Thus, understanding entrepreneurial activity requires attention both to the motivations underlying entrepreneurship and to the type of initiative through which these motivations are expressed. Within this broader picture, gender is particularly relevant, as women’s and men’s entrepreneurial motivations may differ.

### 1.4 Gender differences in motivation for initiatives

Entrepreneurial activity appears to be driven by different motivational patterns among men and women. Studies show that for men, achievement motivation leads directly to entrepreneurial intention, whereas for women, this relationship is primarily driven indirectly through the development of entrepreneurial mindsets and skills (Gupta et al., 2009; Kong and Choo, 2022). Evidence suggests that women are more motivated by a desire for independence and consider their children as motivators, whereas men are more frequently driven by job dissatisfaction (Kirkwood, 2009). Moreover, in many cultures, even women who work are still expected to assume primary responsibility for family and household duties (Mandel and Lazarus, 2021). For many women, a key driver of building one’s own entrepreneurial enterprise may be that it can increase autonomy, thus enabling greater integration between family and career responsibilities (Kimbu et al., 2021; Phillips et al., 2025).

However, women’s entrepreneurial efforts do not merely serve themselves. Indeed, organizations founded by women are key drivers of both the economy and of social service provision (Cardella et al., 2020; Sánchez-Limón et al., 2025). Growing recognition of the importance of diversity and inclusion in achieving economic growth and stability has led to specific governmental and institutional efforts to support women entrepreneurs, although these efforts remain limited in scope and effectiveness (Nziku and Henry, 2020; Simarasl et al., 2024; Xie et al., 2021). While the studies discussed above have substantially advanced understanding of gender differences in entrepreneurial motivations and contextual influences, they have largely examined women entrepreneurs as a relatively homogeneous group. This approach may obscure meaningful differences within women’s entrepreneurship, particularly when the intended beneficiaries of the venture are themselves women. Consequently, less attention has been devoted to understanding whether women who develop ventures specifically for other women are motivated by a distinct configuration of personal, relational, and contextual factors. This group is particularly important to examine because the entrepreneur’s own experiences as a woman may intersect with her recognition of needs experienced by other women, potentially linking self-oriented and other-oriented motivations in ways that are not captured by broader comparisons between women and men entrepreneurs.

### 1.5 Study rational

Despite the growing body of research on women’s entrepreneurship, an important gap therefore remains. Existing studies have largely focused on comparing women and men entrepreneurs or on women entrepreneurs as a broad category. These approaches provide important insights into gender differences in entrepreneurship, but they offer limited understanding of the motivations of women whose ventures are specifically designed to address the needs of other women. This distinction is theoretically important because such ventures may emerge at the intersection of entrepreneurs’ personal experiences, their relationships with others, and their recognition of unmet needs within the wider environment. Examining this subgroup can therefore extend existing accounts of entrepreneurial motivation by identifying how different motivational domains combine within women-centered entrepreneurship. Moreover, much of the existing literature continues to rely on traditional perspectives that do not fully incorporate a gender-based lens (Yadav and Unni, 2016). As argued by Ahl (2006), prevailing assumptions regarding women’s entrepreneurship have often been shaped by gendered and normative frameworks rather than objective differences in entrepreneurial capability, underscoring the need for research that examines women entrepreneurs within the specific social and gendered contexts in which they operate (Ahl, 2006).

### 1.6 Conceptual framework guiding the study: the competency-based framework

The competency-based framework was developed by Agarwal and Agrawal (2023) to explain the process underlying entrepreneurial activity (Agarwal and Agrawal, 2023). The framework includes four constructs: 1. motivational factors for entrepreneurial activity; 2. entrepreneurial learning, which functions as a mediator; 3. competencies, which also function as a mediator; and 4. enterprise performance, which serves as the outcome. The present study focuses on the first construct, namely the motivational factors underlying entrepreneurial activity. This construct encompasses personal drivers (e.g., the desire for work-life balance or the influence of personal life experiences), social drivers (e.g., encouragement from family and friends or the presence of role models), and environmental drivers (e.g., governmental support or cultural characteristics).

The framework was selected because it was developed through a comprehensive synthesis of the literature on women’s entrepreneurship and offers a distinctive perspective on women’s entrepreneurial motivations and experiences. However, it remains grounded primarily in theoretical inquiry and has not yet undergone extensive empirical validation or practical application. Its application to women entrepreneurs whose ventures specifically address the needs of other women therefore provides an opportunity to examine the empirical relevance of its motivational domains while allowing for additional themes and conceptual refinements that may extend or challenge the original framework.

Accordingly, this study aims to explore the motivations of women entrepreneurs who develop initiatives addressing the needs of other women, using the competency-based framework as an initial conceptual lens, and to contribute to the development of an empirically grounded understanding of these motivations.

The remainder of the paper is structured as follows. The next section presents the methodological approach used to explore the motivations of women entrepreneurs developing initiatives for other women, including how the competency-based framework guided the analysis. The findings section then presents how the empirical findings were integrated with the original framework, resulting in the development of the Triple-Driver Framework of Women’s Entrepreneurship, comprising personal, relational, and ecosystem drivers. Finally, the discussion considers the findings and the proposed framework in relation to the existing literature, outlines their theoretical and practical implications, and identifies directions for future research.

## 2 Methodology

This qualitative study employed in-depth, semi-structured interviews with women entrepreneurs whose ventures were specifically dedicated to addressing the needs of other women. The study sought to explore the motivations underlying these initiatives and to develop an empirically grounded understanding of the factors that drive women to establish ventures aimed at improving the lives and opportunities of other women.

### 2.1 Participants and data collection

Between September 2023 and January 2026, 70 women were interviewed for this study. During the interviews, it became apparent that 10 participants were not the original founders of the initiatives they represented and were therefore excluded. The final sample comprised 60 women entrepreneurs. Inclusion criteria included Israeli women aged 18 years and older whose initiatives specifically targeted women’s needs in various topics, such as health, mental health, domestic violence, or culture. Women who were not the founders of the initiative, or whose initiatives did not exclusively target women as the primary population, were excluded from the study. The interview lasted 60 min on average. Participants were recruited using a combination of convenience and snowball sampling. Initially, women entrepreneurs known to the researcher who met the study inclusion criteria were approached and invited to participate. Following each interview, participants were asked to recommend other women entrepreneurs whose initiatives addressed the needs of women and who might be eligible for the study. This snowball sampling process was particularly appropriate given the relatively small and interconnected community of women entrepreneurs developing women-focused initiatives in Israel. Recruitment and interviewing continued until data saturation was reached, as no substantially new themes were emerging from the interviews. At this stage, the same names of potential participants were also repeatedly suggested by different interviewees, further indicating that the relevant participant network had been extensively sampled. Recruitment was therefore concluded after 60 interviews.

The interview guide was developed especially for this study, and addressed the following topics: 1. Family, social, and cultural background- Participants’ childhood environment, gender roles, social norms, challenges of being a woman, and influential female role models; 2. Motivations for the innovation- Understanding the origins of the initiative; 3. Barriers, challenges, and facilitators- Examining gender-related obstacles, difficulties encountered throughout the entrepreneurial journey, and facilitators to overcome them; 4. Opportunities and partnerships- Identifying opportunities and collaborations; and 5. Advice- Insights, lessons learned, and recommendations for other women seeking to promote initiatives for women (see Supplementary Table 1). The current study focused on participants’ motivations for initiating their ventures.

### 2.2 Data analysis

Data was analyzed using explanatory content analysis (Vaismoradi et al., 2013) guided by the conceptual competency-based framework (Agarwal and Agrawal, 2023) described in the Introduction. This approach enabled the researchers to examine participants’ motivations in relation to the framework while remaining open to themes and conceptual refinements emerging from the data.

Microsoft Excel was used to organize and manage the qualitative data throughout the coding and analysis process. The analysis proceeded in four stages. First, both researchers read all interview transcripts in full to become familiar with the data. Second, they independently identified and coded meaning units related to the participants’ motivations for establishing and developing their initiatives. Third, the codes were compared with the personal, social, and environmental drivers proposed in the competency-based framework. Codes that corresponded with the framework were organized under these initial categories, whereas codes that did not fit the original categories were retained and examined inductively. Fourth, the framework-based and emergent codes were integrated by comparing their meanings, boundaries, and relationships across interviews. This process resulted in the retention, refinement, expansion, or relabeling of the original categories according to their ability to explain the participants’ experiences. Specifically, personal drivers were further differentiated into subcategories, social drivers were reconceptualized as relational drivers, and environmental drivers were expanded and reconceptualized as ecosystem drivers.

The analysis was conducted independently by two researchers. The first researcher (LS) holds a PhD and has expertise in social work, public health, mixed-methods research, and qualitative analysis. The second researcher was a social work student who had completed three academic courses in qualitative research and received individual training from LS in explanatory content analysis and in applying the competency-based framework.

Several procedures were used to enhance the trustworthiness of the findings. Both researchers independently coded all interviews and subsequently compared their coding decisions. Discrepancies were examined by returning to the relevant interview excerpts and discussing the interpretation and placement of each code. These discussions continued until consensus was reached. Throughout the analysis, the researchers refined the definitions and boundaries of the categories and subcategories to ensure that they were conceptually coherent, distinguishable from one another, and consistently grounded in the participants’ accounts.

For the purposes of this study, the quotations presented in the Results section were translated from Hebrew into English.

## 3 Results

### 3.1 Overview of main findings

Interviews were conducted with 60 Israeli women entrepreneurs who established ventures aimed at addressing the needs of other women. In addition to the three main drivers of the motivational factors construct of the competency-based framework discussed above, there were also additional themes that extended beyond the original conceptualization. Building on these findings, the present study proposes an expanded model, termed the Triple-Driver Framework of Women’s Entrepreneurship, to capture the experiences and motivations of women entrepreneurs whose initiatives address the needs of other women. While informed by the original distinction between personal, social, and environmental drivers, the new framework reconceptualizes these domains as personal drivers, relational drivers, and ecosystem drivers, and incorporates several new themes and subthemes that emerged inductively from the data.

In this section, I first present the participants’ personal characteristics, followed by the findings related to each of the three domains of the Triple-Driver Framework of Women’s Entrepreneurship.

### 3.2 Participants’ characteristics

A total of 60 women entrepreneurs were included in the study sample. The interviewees were between the ages of 18 and 70 years (mean age = 43.2; median = 41). On average, participants had established their initiatives 6 years prior to the interview (median = 7), with initiatives ranging from less than 1–36 years in operation. 15% of the participants were single, 68% were married or in a relationship, and 17% were divorced or separated. In terms of education, 13% completed certificate studies without obtaining an academic degree, 38% held a bachelor’s degree, 37% held a master’s degree, and 12% held a PhD or MD degree. Most participants identified as Jewish (93%), while 7% identified as Arab. In addition, 35% of the initiatives were for-profit organizations, with the remainder being non-profit organizations. The main areas addressed by the initiatives were health and mental health (45%), employment, economic empowerment, and education (27%), violence, women’s exploitation, and prostitution (13%), political and civic engagement (8%), and women’s identity and culture (7%) (Table 1).

**Table 1:** Participant background characteristics and initiative-related information (*n* = 60).

Characteristic	Participants (*n* = 60)
Age (years: average, median; Min = 18, Max = 70)	43.2, 41
Age group (No., %)	
19–35	11, 18%
36–52	36, 60%
53–70	13, 22%
Years from establishing the initiative (years: average, median; Min = less than a year, Max = 36)	6.0, 7.0
Marital status (No., %)	
Single	9, 15%
Married or in relationship	41, 68%
Divorced or separated	10, 17%
Education (No., %)	
Certificate studies	8, 13%
BA	23, 38%
MA	22, 37%
PhD or MD	7, 12%
Ethnic group (No., %)	
Jewish	56, 93%
Arab	4, 7%
For-profit organization (No., %)	21, 35%
Primary field addressed by the initiative (No., %)	
Health and mental health	27, 45%
Employment, economic empowerment, and education	16, 27%
Violence, women’s exploitation, and prostitution	8, 13%
Political and civic engagement	5, 8%
Women’s identity and culture	4, 7%

### 3.3 The triple-driver framework of women’s entrepreneurship

Building on the competency-based framework of Agarwal and Agrawal (2023), the findings of the present study support the relevance of personal, social, and environmental influences on women’s entrepreneurial motivations while also suggesting several important conceptual refinements. Based on these findings, an expanded model, termed the Triple-Driver Framework of Women’s Entrepreneurship, is proposed. The framework captures the motivations of women entrepreneurs who established initiatives addressing the needs of other women and organizes these motivations into three domains: personal drivers, relational drivers, and ecosystem drivers. Figure 1 presents the framework and its main themes and subthemes.

**Figure 1:**
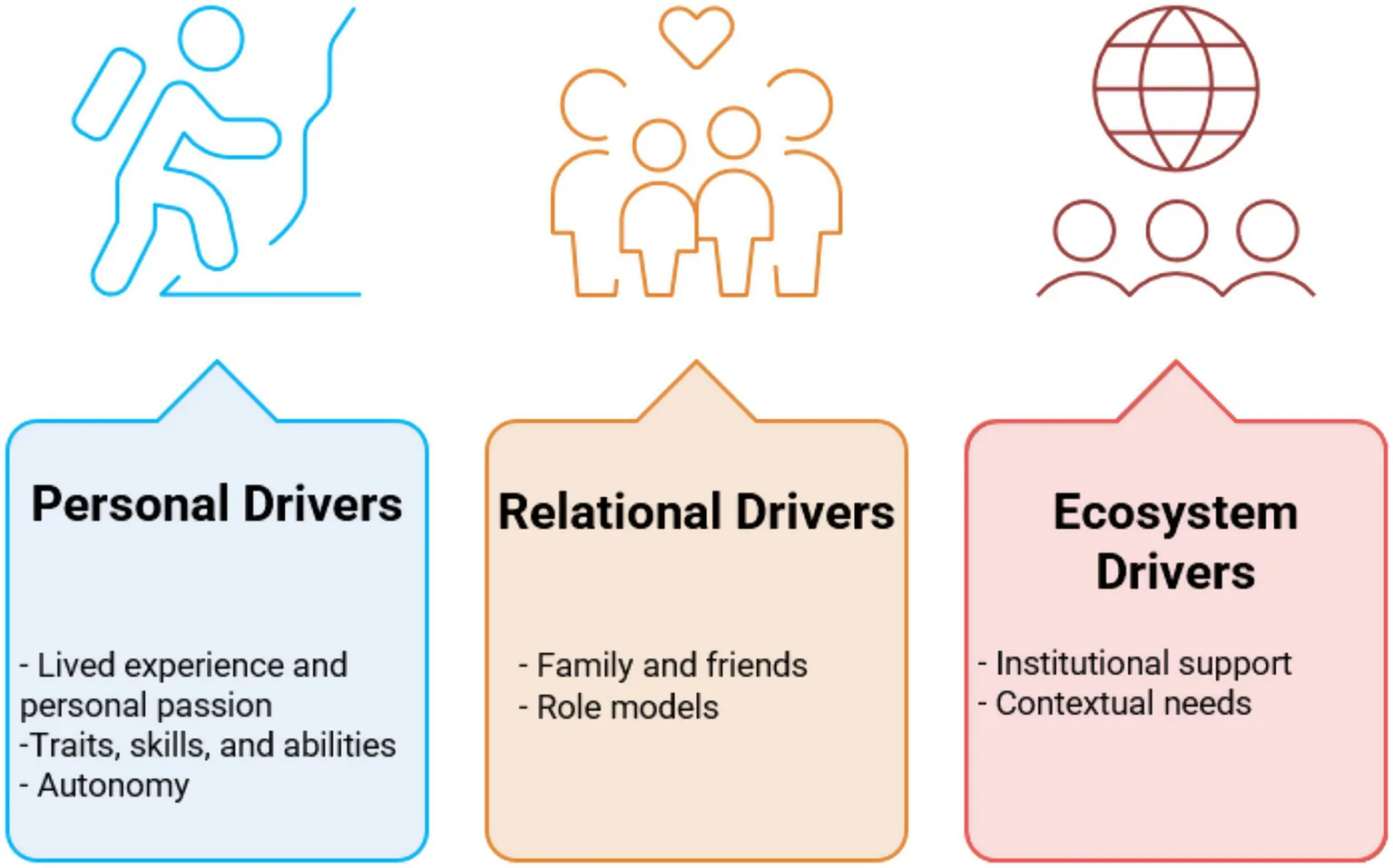
The triple-driver framework of women’s entrepreneurship.

### 3.4 Integration of the original framework and empirical findings

The analysis presented below demonstrates how the original competency-based framework was integrated with the empirical findings to construct the Triple-Driver Framework of Women’s Entrepreneurship. The original domains of personal, social, and environmental drivers served as the initial structure for organizing and interpreting the data. Within each domain, participants’ accounts were examined to determine which components of the original framework were supported, which required expansion or reconceptualization, which were not sufficiently represented in the interviews, and which additional motivational components emerged from the data.

Accordingly, each section first describes the relevant domain of the original framework and then explains how the findings supported, extended, or modified its conceptualization. This analytical process resulted in the expansion of the personal drivers domain, the reconceptualization of social drivers as relational drivers, and the broadening of environmental drivers into ecosystem drivers. Although the three domains are presented separately for analytical clarity, they are not mutually exclusive, as individual participants could describe motivations associated with more than one domain. The following sections present this integration and the empirical basis for each component of the proposed framework.

## 4 Personal drivers

The personal drivers domain constitutes the first component of the Triple-Driver Framework of Women’s Entrepreneurship. In the original framework, personal drivers encompass motivations related to work-life balance, educational background, and personal experiences that inspire women to initiate entrepreneurial ventures (Agarwal and Agrawal, 2023). The findings of the current study extend this conceptualization by suggesting that personal skills, abilities, values, and personality traits, as well as a broader desire for autonomy and self-determination, may serve as important motivational forces underlying entrepreneurial activity. In contrast, educational background was rarely mentioned by the interviewees and, therefore, was not retained as a component of the Triple-Driver Framework of Women’s Entrepreneurship.

### 4.1 Lived experience and personal passion

Agarwal and Agrawal (2023) conceptualize personal experiences as a motivational driver, referring to entrepreneurs’ life experiences that inspire them to initiate entrepreneurial ventures. Consistent with this framework, personal experiences emerged as a central source of motivation among the women entrepreneurs in the current study. For example, an entrepreneur working in the field of domestic violence described how growing up in a violent environment and personally experiencing violence later motivated her entrepreneurial activity.

At the same time, the findings suggest that this category should be broadened. Specifically, 44 of the 60 entrepreneurs reported motivations that stemmed either from their own lived experiences and past struggles, or from a longstanding passion and personal commitment to the issue they sought to address- even if they had not personally experienced it. Consequently, this study expands the original personal experiences driver into a new category, termed lived experience and personal passion. While lived experiences align with the original conceptualization proposed by Agarwal and Agrawal (2023), enduring passion and personal interest represent a distinct yet closely related source of motivation that was not explicitly captured in the initial framework.

For instance, an entrepreneur promoting awareness of genetic diseases within her ethnic community explained that her initiative was not driven by her own experience per se, but rather by her deep commitment to empowering women and improving community health. These findings indicate that entrepreneurial motivation may arise not only from personal experiences but also from sustained passions and commitments directed toward addressing women’s needs, thereby broadening the original understanding of experience-based motivation.

To preserve participants’ anonymity and confidentiality, no illustrative quotations are presented in this section, as the unique nature of some experiences could potentially lead to participant identification.

### 4.2 Traits, skills, and abilities

Initially, this driver was not mentioned in the original conceptual framework. However, interviewees were asked which personal traits, skills or abilities served as drivers for the initiative. The entrepreneurs mentioned various answers, and they were categorized into five major themes which will be presented in the following section.

#### 4.2.1 Values of justice and correcting social injustices

One major trait that was mentioned often was a sense of justice, and the desire to address the wrong. This trait was mentioned by 29 interviewees. Here are some examples for quotes:

“I have a very, very strong sense of justice. When I see injustice in front of me, it really upsets me, deeply enrages me, and motivates me to act… When something happens, I fight back, and I really struggle against it… my defense mechanism against trauma is very much engaging in activism.” (Entrepreneur in her 20s in the field of political and civic engagement).

“I channeled it into entrepreneurship and very intensive activism, because my sense of justice became extremely developed. I could not stand by when I saw injustice… I refused to conform to certain norms or conventions. I was constantly fighting.” (Entrepreneur in her 50s in the field of violence, women’s exploitation, and prostitution).

#### 4.2.2 Interpersonal abilities

Another personal characteristic identified in the study was a set of interpersonal abilities, including empathy, attentiveness and sensitivity to others, and a desire to empower others. This theme was mentioned by 22 of the 60 interviewees. The following examples illustrate how these interpersonal qualities were expressed in the interviews:

“I truly understand people… I felt that I had a really good connection with patients because I genuinely relate to them at eye level. I think that this is what gave me the drive to do what I do.” (Entrepreneur in her 30s in the field of health and mental health).

“People have always fascinated me more than anything else… I often feel that I understand the misunderstandings that arise between people and that I can help bridge those gaps. I want to bring a little light into the conflict.” (Entrepreneur in her 40s in the field of violence, women’s exploitation, and prostitution).

#### 4.2.3 Leadership, organizational management, and entrepreneurial skills

Another relatively prevalent personal characteristic, mentioned by 22 of the 60 interviewees, related to their skills in the areas of leadership, organizational management, and entrepreneurship. This theme encompassed qualities such as charisma and the ability to inspire and lead others, practical competencies required to manage and advance an initiative, and entrepreneurial attributes, including the courage and determination to pursue a vision even when others do not yet recognize its potential. The following examples illustrate this theme:

“The fact that I worked in high-tech means that I really know how to take a dream and turn it into something concrete and actionable. I’m also very strong [at executing a plan]… I never actually studied anything specifically for this.” (Entrepreneur in her 30s in the field of employment, economic empowerment, and education).

“I think I’m very courageous… I’m extremely courageous, and I can make decisions very quickly… I think people are drawn to energy… That’s the kind of feedback I receive… you could call it contagious energy.” (Entrepreneur in her 50s in the field of health and mental health).

#### 4.2.4 Determination and resilience competencies

Another relatively prevalent theme, mentioned by 21 of the 60 interviewees, concerned personal qualities such as determination, optimism, resilience in coping with challenges, and the ability to navigate uncertainty- characteristics that are often essential in entrepreneurial endeavors. Participants described these capabilities as important factors that motivated and enabled them to establish their initiatives. The following quotations illustrate this theme:

“Determination, complete self-belief, and a kind of blindness to limitations and obstacles… When you look at the world, do you see open doors or closed ones? I have a certain blindness to limitations and barriers, so I only see open doors… I simply walk through them.” (Entrepreneur in her 40s in the field of health and mental health).

“Entrepreneurship involves a kind of irrepressible optimism, almost to the point of foolishness [laughs]… We can go to bed feeling discouraged and frustrated, and then wake up the next morning to a new day, full of energy… I strongly believe in running a marathon rather than a sprint, and I have that ability- patience combined with entrepreneurship and a sense of urgency.” (Entrepreneur in her 50s in the field of employment, economic empowerment, and education).

#### 4.2.5 Creativity, innovativeness, curiosity, and learning attributes

Creativity, innovativeness, curiosity, and a strong orientation toward learning also emerged as relatively prevalent personal characteristics. This theme was mentioned by 21 of the 60 interviewees. The following examples from the interviews illustrate these attributes:

“I’m also a very curious person. If there’s a topic that interests me, I’ll dive deeply into it and learn everything I can about it… That kind of obsession with the things you love, that curiosity, is an incredibly valuable trait in entrepreneurship because you constantly have to acquire new skills and learn things you never knew before.” (Entrepreneur in her 20s in the field of violence, women’s exploitation, and prostitution).

“I’m much more entrepreneurial by nature. I’m the kind of person who comes up with ideas and new ways of thinking… My stronger side is thinking, often outside the box, creatively, and not according to conventional patterns. I come up with ideas that no one else thinks of… I stay at the idea stage and then move on to the next idea.” (Entrepreneur in her 50s in the field of employment, economic empowerment, and education).

### 4.3 Autonomy

Another personal driver identified in Agarwal and Agrawal’s (2023) framework is work-life balance. This motivation refers to entrepreneurs’ desire to gain greater control over balancing professional responsibilities with family demand (Agarwal and Agrawal, 2023). However, in the current study, this theme was mentioned by only one interviewee- a woman in her 30s working in the field of women’s identity and culture, who explained that her initiative emerged from her need to better manage the challenges of new motherhood while maintaining her professional aspirations:

“…my eldest daughter was born. Then my world collapsed, because I realized that I could no longer continue doing what I had been doing, since I could not leave for ten days at a time [for work].… it did not suit me.”

Interestingly, three additional interviewees expressed a desire to be their own bosses and to have greater control over their work decisions, schedules, and methods. The following two quotes illustrate this motivation:

“I’ve never really fit well within structured frameworks, so it was always clear to me that I would need to run my own business. I did not necessarily think it would be a start-up. I just thought I would establish a business, but it eventually developed in that direction.” (Entrepreneur in her 20s in the field of violence, women’s exploitation, and prostitution).

“So, I’ll say that I’m a terrible employee. In every place I worked, I eventually got tired of being told what to do. So, I also needed to find a solution for not being an employee anymore, and that became a drive as well.” (Entrepreneur in her 30s in the field of employment, economic empowerment, and education).

Building on the original framework, the findings suggest a broader motivational construct of autonomy, encompassing both work-life balance and work autonomy. This expanded concept reflects entrepreneurs’ aspirations to balance family and professional responsibilities while maintaining independence and control over their work decisions, schedules, and methods. Accordingly, autonomy constitutes the third component of the personal drivers domain within the Triple-Driver Framework of Women’s Entrepreneurship.

## 5 Relational drivers

Agarwal and Agrawal (2023) originally conceptualized the domain of social drivers, arguing that entrepreneurship enables women to gain recognition, status, and an independent identity by fostering confidence, self-worth, visibility, and distinct personal and professional identities within society. Their framework also identifies family, friends, and role models as important sources of social motivation. However, the findings of the current study did not provide evidence that social recognition, status, or identity formation served as primary motivations for establishing women-centered ventures. Instead, participants’ motivations were rooted primarily in specific relationships and influential figures in their lives rather than in society at large. I therefore reconceptualize this domain as “Relational Drivers.” Although many interviewees described meaningful female role models from their childhood and adolescence, only a few explicitly identified these role models as direct motivations for initiating their ventures. Similarly, only a limited number of participants referred to the support and encouragement of family members and friends as motivating factors. Accordingly, this domain was reconceptualized as relational drivers in the Triple-Driver Framework of Women’s Entrepreneurship, emphasizing the influence of significant relationships and role models on women’s entrepreneurial motivations. The following section presents the findings related to these relational sources of motivation.

### 5.1 Family and friends

Support from family and friends was mentioned only minimally throughout the interviews by three interviewees. The following quotation illustrates this theme:

“There was really a sense in my family that I wasn’t supposed to hold myself back or try to please everyone… a very liberal environment that encouraged independence and independent thinking… My parents also ran their own company for many years… There’s also a somewhat rebellious nature in my family, and difficulty with authority, and that was something that was supported and reinforced at home.” (Entrepreneur in her 30s in the field of health and mental health).

“Violence against my mother was part of my childhood. I saw her suffering, but I could not help her… As I grew older, I met women living in shelters through my work, and when I heard their stories, I began thinking about what I could do for them… I started thinking about women affected by violence who leave shelters without the tools to return to independent living, just like my mother… That is why I began developing my initiative, which is intended for women who do not know how to start an independent life after leaving the shelter.” (Entrepreneur in her 50s in the field of violence, women’s exploitation, and prostitution).

### 5.2 Role model

Women in the study were also asked about a significant female figure they remembered from childhood or adolescence. A total of 40 interviewees mentioned role models, including family members such as mothers, grandmothers, or aunts, while others referred to public figures such as politicians or authors. However, only two of them explicitly identified these figures as motivators for initiating their entrepreneurial activity. The following quotations illustrate this theme:

“The mentor I worked with on the project is one of the most impressive women I’ve ever met… Seeing her speak with so much self-confidence gave me a huge amount of confidence too, and helped me realize that I could also be like that- stand in front of people, and if there’s something I want to promote, I’ll promote it.” (Entrepreneur in her late teens in the field of violence, women’s exploitation, and prostitution).

“A fifth-grade teacher whom I remember very well, especially in relation to my professional path, had a profound influence on me. She was my homeroom teacher, and she took it upon herself to teach healthy sexuality in a way I had never encountered before. She made it the focus of an entire school year.” (Entrepreneur in her late teens in the field of health and mental health).

Notably, 16 interviewees stated that they did not recall any significant figure from their childhood or adolescence. Two interviewees explicitly mentioned that this lack of a role model in their own lives was in part a driver for their own initiatives. The following quotes exemplify this:

“That’s my story. I did not have a figure like that [laughs]. I became the figure that I needed… I think that today, I create and act from a place of absence, of lacking. From what I was missing.” (Entrepreneur in her 20s in the field of women’s identity and culture).

“I did not want to be like my mother. In a way, that was my motivation to do things differently. I did not want to be like her in the sense of being a woman who depends on a man for everything-financially, for transportation, for all kinds of things…” (Entrepreneur in her 40s in the field of employment, economic empowerment, and education).

It is worth noting that the interviewees were not specifically asked whether their role models had served as a motivation for initiating their ventures, nor were they asked about role models in general, as the interview guide focused exclusively on female role models. It is possible that a different line of questioning would have elicited additional responses on this topic.

## 6 Ecosystem drivers

Agarwal and Agrawal (2023) conceptualized the domain of environmental drivers, referring to external factors that motivate individuals to initiate entrepreneurial ventures. Their framework includes institutional actors, such as government bodies and organizations, which provide support through education, funding, information, and knowledge, as well as cultural values and characteristics that encourage women’s entrepreneurial activity (Agarwal and Agrawal, 2023). The findings of the current study support these dimensions while also suggesting a broader conceptualization of “Ecosystem Drivers.” Beyond institutional support and cultural influences, participants frequently described their recognition of unmet societal and contextual needs as a key source of motivation for establishing their initiatives. In many cases, women were driven to act after identifying gaps in existing services, resources, or opportunities available to other women. Consequently, the term ecosystem drivers more accurately captures the range of external influences identified in the data, encompassing institutional support, cultural contexts, and the recognition of societal needs that motivate entrepreneurial action. The following section presents the findings related to these ecosystem drivers.

### 6.1 Institutional support

Eight interviewees in the study mentioned 11 institutions that served as motivators for initiating their ventures (three mentioned two institutions). These included academic programs abroad, pre-academic programs, NGOs, and similar frameworks. The following excerpts from the interviews illustrate this theme:

“I completed my internship and traveled to Boston for a social entrepreneurship program for Israelis and Palestinians. That’s where I met my team, and we decided to establish the organization as a non-profit.” (Entrepreneur in her 30s in the field of health and mental health).

“We started at the Michal Sela Forum [NGO] in the field of domestic violence. We basically wanted to help women trapped in the cycle of violence… I participated in the first hackathon held, and I really connected with the founder of the forum. Together, we went through a process involving the Ministry of Welfare and various centers for the treatment and prevention of domestic violence.” (Entrepreneur in her 20s in the field of violence, women’s exploitation, and prostitution).

### 6.2 Contextual needs

Originally, Agarwal and Agrawal (2023) conceptualized the domain of “contextual needs” as the influence of cultural characteristics that motivate individuals to engage in entrepreneurial activities (Agarwal and Agrawal, 2023). In the current study, none of the interviewees identified cultural aspects as direct motivators for initiating their ventures. However, 44 of the 60 interviewees described cultural and gender-related gaps in their environments that encouraged them to take action and establish their initiatives. The following quotes illustrate this theme:

“After conducting deeper research and reading more on the topic, we said that we had to do something with it… It’s impossible that women are saying so many things, yet no one talks to us about it- not doctors, not partners, not family, not friends. No one… That’s why we decided to establish [the initiative].” (Entrepreneur in her 20s in the field of health and mental health).

“In the area of money and investments, women have a kind of existential fear that is connected to the way we were raised and to what society expects from us… society does not expect women to be brave in these areas. In the end, women earn less than men, so they also have less money to risk in investments… As a result, women become so protective and so risk-averse that they simply remain behind… So what led me to act on behalf of women in this field was precisely seeing these gaps” (Entrepreneur in her 50s in the field of employment, economic empowerment, and education).

## 7 Discussion

The aim of this study was to explore the motivations of women entrepreneurs who dedicated their initiatives to addressing the needs of other women. In total, 60 women entrepreneurs participated in in-depth interviews, and the data were analyzed using explanatory content analysis. The analysis was guided by the competency-based conceptual framework developed by Agarwal and Agrawal (2023), with a particular focus on its motivational factors construct (Agarwal and Agrawal, 2023). However, the findings of the present study extended the original framework by incorporating insights derived from empirical data, resulting in the development of the Triple-Driver Framework of Women’s Entrepreneurship. This framework comprises three interconnected domains: personal drivers, relational drivers, and ecosystem drivers.

The two most prevalent personal drivers identified in the present study were lived experience and personal passion, and a commitment to social justice and the rectification of injustices. Women entrepreneurs who establish initiatives for other women are often motivated by strong personal experiences and deeply held values (Solesvik et al., 2019). These experiences and values are transformed into collective action and a desire to give back to their communities (Meyer et al., 2022). Previous research has similarly demonstrated that women’s entrepreneurial activities are frequently driven by moral commitments, solidarity, and a desire to create social value for others rather than solely by economic considerations (Fernández-Guadaño and Martín-López, 2023; Lenz et al., 2025). In particular, women entrepreneurs often engage in helping behaviors grounded in ethical responsibility, care, and community empowerment, regardless of their educational background (Peake and Eddleston, 2021). The combination found in the current study of personal experiences with a commitment to social justice may therefore foster a generative orientation- an enduring motivation to use one’s knowledge, resources, and experiences to support others and promote broader social change. Such findings suggest that, for many women entrepreneurs, venture creation represents not only an individual career path but also a mechanism for transforming personal adversity and values into meaningful contributions to the lives of other women. Although these motivations were categorized as personal drivers within the proposed framework, they reflect both self-oriented and other-oriented forms of motivation, and in many cases, a blending of the two.

The motivations reported by participants reflected a combination of self-oriented, other-oriented, and blended patterns. Some motivations were primarily other-oriented, stemming from a desire to help and empower other women, promote social justice, address unmet societal needs, or tackle issues that participants had not personally experienced but nevertheless sought to change. Other-oriented motivation refers to the aspiration to create positive social impact and respond to the needs of individuals and communities (Yahyaoui et al., 2023). In contrast, several participants expressed predominantly self-oriented motivations, such as a desire to address challenges they had personally encountered or to achieve greater autonomy and control over their professional lives. The finding that more than one-third of the initiatives operated as for-profit ventures further highlights the importance of personal aspirations and intrinsic motivations, which characterize self-oriented entrepreneurial behavior (Mascena Barbosa and Dumont, 2024). However, many entrepreneurs exhibited blended motivations, combining self-oriented and other-oriented goals. These women transformed personal experiences, interests, and aspirations into initiatives designed to benefit other women and society more broadly. Such a pattern aligns with the concept of blended motivation, in which entrepreneurial activity is simultaneously driven by personal fulfillment and a commitment to creating social value (Fauchart and Gruber, 2011).

Beyond the categorization of entrepreneurial motivations, the findings of the current study highlight the prominent role of personal traits, skills, and abilities as motivational drivers for venture creation. In other words, because these women realized that they had particular talents and capabilities, they felt called to use them somehow to promote the greater good. Although this dimension is not explicitly included in the competency-based framework proposed by Agarwal and Agrawal (2023), it emerged as a central theme across participants’ narratives. Besides the commitment to justice and the rectification of social injustices, interpersonal qualities such as empathy and a desire to empower others have been linked to women’s orientation toward social value creation (Miller et al., 2012). A 2022 study examining the relationship between empathy and social entrepreneurial intention among 250 aspiring social entrepreneurs found that this association was mediated by social entrepreneurial self-efficacy (Kim, 2022). In the current study, interpersonal qualities as well as personal live events were also major factors that motivated the entrepreneur. Interestingly, there is a link between empathy and life events. A 2022 Belgian qualitative study of 33 social entrepreneurs explored how empathy shapes entrepreneurial motivations and helps entrepreneurs navigate tensions between social and commercial goals. The findings showed that entrepreneurs driven by empathy often traced their motivation to critical life experiences.

At the same time, although social impact was a central objective, some participants emphasized that financial sustainability was equally or even more important for their organization (Lambrechts et al., 2020). Their emphasis on financial sustainability may reflect the broader financial challenges associated with entrepreneurial activity, as financial constraints have been identified as a major barrier to establishing and sustaining entrepreneurial ventures (Halim et al., 2023). These findings suggest that interpersonal qualities, such as empathy, influence entrepreneurial activity through other personal characteristics, including self-efficacy and other life experiences. The current study extends this literature by demonstrating that interpersonal abilities are a central motivational driver among women entrepreneurs whose ventures address the needs of other women, regardless of whether their initiatives operate as for-profit or nonprofit organizations.

Building on the previous finding that interpersonal qualities may motivate entrepreneurial activity through entrepreneurial self-efficacy, the current study further suggests that women’s perceptions of their own leadership and entrepreneurial capabilities can themselves serve as motivational drivers for venture creation. Participants frequently described their ability to lead others, manage organizations, make decisions, and transform ideas into action as reasons they felt capable of initiating their ventures. This finding aligns with the entrepreneurship literature, which distinguishes entrepreneurial competencies from entrepreneurial self-efficacy while recognizing that confidence in one’s entrepreneurial capabilities strengthens entrepreneurial intentions and increases the likelihood of venture creation (McGee et al., 2009; Nowiński et al., 2019). In addition, determination, optimism, resilience in coping with challenges, and the ability to navigate uncertainty, emerged as central motivational forces. Given that entrepreneurship inherently involves ambiguity, setbacks, and risk, these psychological resources have consistently been associated with entrepreneurial persistence and long-term venture engagement (Adomako et al., 2016; Hartmann et al., 2022). The present findings extend this literature by suggesting that leadership, entrepreneurial competencies, and resilience function not only as attributes that facilitate entrepreneurial success but also as intrinsic motivations that encourage women to establish ventures dedicated to addressing the needs of other women.

The prominence of personal and contextual drivers identified in the current study stands in contrast to the minimal role attributed to institutional support in motivating entrepreneurial activity. Although the entrepreneurship literature has increasingly emphasized the importance of external support systems for women entrepreneurs, including governmental policies, entrepreneurship education, incubators, financial institutions, and nonprofit organizations (Adhikari, 2025; Brush et al., 2018; Serpente et al., 2025), such sources of support were rarely mentioned by participants as motivations for establishing their ventures. Notably, none of the interviewees identified governmental support as a motivating factor, and only a few referred to nongovernmental organizations or educational institutions. These findings may suggest that existing institutional support mechanisms are not sufficiently visible, accessible, or perceived as relevant by women during the early stages of venture creation. Alternatively, they may reflect a continued lack of effective institutional support for women entrepreneurs. That is, the women in the current study may have succeeded without the support of such support, which is why so few of them mentioned it as an important factor. Although the present study cannot distinguish between these explanations, the findings highlight a potential gap between the increasing policy emphasis on supporting women’s entrepreneurship and entrepreneurs’ lived experiences. Strengthening institutional support systems and improving their visibility and accessibility may therefore serve not only to enhance the long-term sustainability of women-led ventures but also to motivate more women to initiate entrepreneurial activity (Lopez et al., 2026; Mwesigwa et al., 2025). Consequently, while recent policy initiatives have increasingly recognized the importance of strengthening institutional ecosystems for women entrepreneurs, the present findings highlight the continued centrality of intrinsic motivations in driving women to create ventures for other women. Given the limited institutional influence reported in this study, strengthening peer support networks and communities of women entrepreneurs may represent a particularly valuable strategy for fostering women-centered entrepreneurship, as such networks have been shown to enhance entrepreneurial confidence, resource sharing, and long-term venture sustainability (Abdul Malik et al., 2025; Lenz et al., 2025). This study has several limitations that should be acknowledged. First, the study was conducted within the Israeli context, and the applicability of the findings in other cultural contexts may be limited. Nevertheless, the relatively large sample of 60 women entrepreneurs representing diverse initiatives and backgrounds enhances the credibility and richness of the findings. Second, the study employed a qualitative design based on semi-structured interviews. Although this approach enabled participants to describe their experiences and motivations in depth and in their own words, it is possible that alternative interview questions or prompts would have elicited additional or different insights. Third, some participants reflected on ventures they had established more than two or three decades earlier, introducing the possibility of recall bias. To minimize this limitation, the interviews were designed to encourage participants to recount their life stories chronologically, beginning with their childhood and progressing through key life experiences before discussing the development of their ventures. This life-history approach was intended to facilitate more accurate recollection of the experiences and motivations that shaped their entrepreneurial journeys. Finally, the proposed Triple-Driver Framework of Women’s Entrepreneurship was developed inductively from qualitative data and should therefore be considered an empirically grounded conceptual model that warrants further validation through quantitative research and across different cultural and institutional contexts. Comparative studies could also examine whether the motivations of women entrepreneurs whose ventures address the needs of other women differ from those of women entrepreneurs serving broader populations. An additional direction for future research is to examine the relationships among the personal, relational, and ecosystem drivers identified in the framework. While the present study focused on identifying and conceptualizing these three sets of motivational drivers, future studies could explore whether and how they interact, reinforce one another, or vary in their relative influence on entrepreneurial motivation. Such research could further clarify whether particular combinations or patterns of drivers characterize different entrepreneurial experiences and contribute to the further development and validation of the Triple-Driver Framework.

## 8 Conclusion

This study contributes to the women’s entrepreneurship literature by providing an empirically grounded understanding of the motivations of women entrepreneurs who establish ventures dedicated to addressing the needs of other women. Building on the competency-based framework of Agarwal and Agrawal (2023), and based on the findings of this study, I propose the Triple-Driver Framework of Women’s Entrepreneurship. The Triple-Driver Framework expands the original conceptualization by identifying three interrelated domains of motivation: personal drivers, relational drivers, and ecosystem drivers. The findings demonstrate that women’s entrepreneurial motivations extend beyond personal experiences to include deeply held values, interpersonal qualities, leadership and entrepreneurial capabilities, resilience, and the recognition of unmet societal needs. By offering a refined conceptual framework grounded in women’s lived experiences, this study advances the theoretical understanding of women-centered entrepreneurship and provides practical insights for researchers, policymakers, and organizations seeking to foster initiatives that improve the lives of women. From a practical perspective, the Triple-Driver Framework provides policymakers, professionals, and organizations with a structured way to understand the different sources of women’s entrepreneurial motivation. This understanding may inform support programs that consider not only individual motivations but also relational and ecosystem-level influences. For women entrepreneurs themselves, recognizing these drivers may help clarify the factors shaping their entrepreneurial actions and more intentionally direct and develop their initiatives.

## Data Availability

The raw data supporting the conclusions of this article will be made available by the authors, without undue reservation.
