# Supplementary material for: From need to action: exploring the motivations of women entrepreneurs through the triple-driver framework of women’s entrepreneurship
Source: Front Psychol. 2026 Sep 29;17:1932247. doi: 10.3389/fpsyg.2026.1932247 (PMC13657938; doi:10.3389/fpsyg.2026.1932247)
Supplement: Supplementary file 1 [file Table_1.DOCX]

# supplementary 1: Interview Guide

| Topic | Question |
| --- | --- |
| Opening | 1. Please tell me a little about yourself and what your childhood was like. |
| Understanding the family, social, and cultural context in which the participant grew up | 2. How would you describe the environment in which you grew up (family, neighborhood, village/community)?  3. Looking back at the environment in which you grew up, how were women characterized in comparison to men in terms of roles at home, employment, informal norms, and expectations?  4. How was a woman who wanted to look different, dress differently, or engage in a field considered unconventional perceived by people in the environment in which you grew up?  5. In your opinion, what was the most challenging aspect of being a woman in the environment in which you grew up (family, neighborhood, village/community)?  6. Is there a significant female figure you remember from your childhood or adolescence? Please explain why |
| Motivations for innovative initiatives for women | 7. Please tell me a little about your innovative initiative for women.  8. What motivated you to act on behalf of women in the field of [specify the field addressed by the innovation]?  9. Was there a specific event or experience that influenced your decision to engage in the field of [specify the field addressed by the innovation]?  10. What personal characteristics do you believe led you to engage specifically in innovation within this field?  11. The next question may be somewhat generalizing, but I would still like to ask it. If your initiative had been led by a man rather than a woman, in what ways do you think it would have been managed differently? |
| Barriers in pursuing innovative initiatives for women | 12. What barriers or difficulties have you encountered in the past, or even today, in working in the field of [specify the field addressed by the innovation], specifically because you are a woman?  13. How did you manage to overcome these barriers or difficulties?  14. What personal costs, if any, were associated with your work on behalf of women? |
| Opportunities | 15. What opportunities emerged along your path while working on behalf of women in the field of [specify the field addressed by the innovation]?  16. Which partners or collaborators emerged along your path while working on behalf of women in the field of [specify the field addressed by the innovation]? |
| Conclusion | 17. What advice would you give to other women who wish to break barriers and create change for other women? |
